# Discriminant Profiles of Volatile Compounds in the Alveolar Air of Patients with Squamous Cell Lung Cancer, Lung Adenocarcinoma or Colon Cancer

**DOI:** 10.3390/molecules26030550

**Published:** 2021-01-21

**Authors:** Leonardo Politi, Lorenzo Monasta, Maria Novella Rigressi, Andrea Princivalle, Alessandro Gonfiotti, Gianna Camiciottoli, Luigi Perbellini

**Affiliations:** 1 Department of Clinical and Experimental Medicine, Careggi University Hospital, 50134 Florence, Italy; leonardo.politi@unifi.it (L.P.); marianovella.ringressi@unifi.it (M.N.R.); agonfiotti@gmail.com (A.G.); gianna.camiciottoli@unifi.it (G.C.); 2 Institute for Maternal and Child Health—IRCCS Burlo Garofolo, 34137 Trieste, Italy; 3 Occupational Medicine, Department of Diagnostics and Public Health, University of Verona, 37134 Verona, Italy; andrea.princivalle@univr.it (A.P.); luigi.perbellini@univr.it (L.P.)

**Keywords:** volatile compounds, alveolar air, squamous cell lung cancer, lung adenocarcinoma, colon cancer, ion-molecule reaction mass spectrometry (IMR-MS)

## Abstract

The objective of the present work was to analyze volatile compounds in alveolar air in patients with squamous cell lung cancer, lung adenocarcinoma or colon cancer, to prepare algorithms able to discriminate such specific pathological conditions. The concentration of 95 volatile compounds was measured in the alveolar air of 45 control subjects, 36 patients with lung adenocarcinoma, 25 patients with squamous cell lung cancer and 52 patients with colon cancer. Volatile compounds were measured with ion molecule reaction mass spectrometry (IMR-MS). An iterated least absolute shrinkage and selection operator multivariate logistic regression model was used to generate specific algorithms and discriminate control subjects from patients with different kinds of cancer. The final predictive models reached the following performance: by using 11 compounds, patients with lung adenocarcinoma were identified with a sensitivity of 86% and specificity of 84%; nine compounds allowed us to identify patients with lung squamous cell carcinoma with a sensitivity of 88% and specificity of 84%; patients with colon adenocarcinoma could be identified with a sensitivity of 96% and a specificity of 73% using a model comprising 13 volatile compounds. The different alveolar profiles of volatile compounds, obtained from patients with three different kinds of cancer, suggest dissimilar biological–biochemistry conditions; each kind of cancer has probably got a specific alveolar profile.

## 1. Introduction

The presence of thousands of substances produced by the human body and detectable in the exhaled (and alveolar) air, together with the use of increasingly sensitive and specific analytical tools, opens up a field of study that many researchers consider with growing interest. Research projects are allowing the deepening of physiological biochemical and metabolic aspects, but also help the study of diagnostic and pathophysiological aspects of many diseases.

Boots et al. [1] published a comprehensive critical review about volatile organic compounds (VOCs) in breath; they framed the multiple issues around breath sampling, discussing the limits and advantages of different analytical techniques used for VOC analysis in exhaled air. The same authors have stated that VOCs in exhaled air can give information about oxidative stress, which is associated with the pathophysiology of several chronic diseases, including sarcoidosis, idiopathic pulmonary fibrosis, chronic obstructive pulmonary diseases, inflammatory bowel diseases and cardiovascular diseases. Moreover, infectious diseases and different kinds of cancer can be detected through the analysis of exhaled VOCs.

Among all tumors, lung cancer currently has the highest rates of mortality in the world [2]. In the United States, about 234,000 new cases of lung cancer are diagnosed each year, representing 13% of all cancer diagnoses, while 154,000 US citizens were expected to die from lung cancer in 2018, accounting for approximately 25% of all cancer deaths [3]. In Italy, 41,000 new cases are expected every year [4]. Early diagnosis of cancer diseases represents the cornerstone of an effective treatment and functional recovery. It is, thus, essential to develop better screening tests that work with biological matrices and can be carried out non-invasively.

Colon cancer has a high incidence and mortality in the world, being the third most common malignant tumor and a major cause of worldwide cancer morbidity and mortality [5]. In the USA, there are more than one million people with a diagnosis of colon-rectum cancer, and approximately 143,000 new cases of colon–rectum cancer were recorded in 2012. Only 59% of men and women aged 50 years and older undergo colorectal cancer screening, and only 39% of patients are diagnosed at an early stage, when treatment is most successful [6]. Estimated numbers of cancer cases in the 40 European countries set colon cancer in the second position (incidence in 2018: 500,000 cases). Deaths due to colon cancer rank second (234,000 deaths), right after the deaths for lung cancers [7].

Over 30 years ago, Gordon et al. [8], through a pioneering method that used 40 L of exhaled air, reported that patients with pulmonary neoplasms had different concentrations of specific VOCs than control subjects. Following this study, many other research projects have been performed with patients with varying kinds of cancers, using more and more sophisticated analytical instruments and statistical elaborations, but with increasingly effective and sometimes even faster diagnostic perspectives.

More recently, some studies confirmed that colorectal cancers also modify the physiological status of patients, together with the breath profile of VOCs: some VOCs are increased, while others decrease if compared to those measured in control subjects [9,10].

Breath samples contain hundreds of compounds that have been commonly measured by different authors in recent decades; many volatile compounds have been identified from the breath of patients with lung cancer, which is the most studied since 1985 [8,11], or breast cancer [12]. Increasingly popular research projects in the literature show that breath tests are useful in different pathological conditions, like esophageal and gastric adenocarcinomas [13], colorectal cancers [14], cervical cancers [15], ovarian cancers [16] and pancreas adenocarcinomas [17,18]. Additionally, chronic diseases, such as Crohn’s disease or ulcerative colitis [19,20,21], cystic fibrosis [22], chronic obstructive pulmonary diseases [23] and different infective diseases [24] show changes in the VOC pattern.

To date, all studies have tried to isolate the pattern of “most” typical molecules produced in such conditions, thanks to the good sensitivity of detectors and an equally refined statistical interpretation and discrimination of collected data. However, results differ depending on the different working groups. Critical issues have arisen [25] because of the different techniques of sample collection (all the exhaled breath or only the alveolar portion, one single breath or a consistent volume of breath collection) and various equipment used: gas chromatography–mass spectrometry analysis (GC-MS), proton transfer reaction–mass spectrometry (PTR-MS), selected ion flow tube–mass spectrometry (SIFT-MS), ion molecule reaction–mass spectrometry (IMR-MS), tests for electrical conductivity (e-nose), colorimetric sensor array and gold nanoparticle sensors. In recent decades, the use of GC-MS has allowed for identifying several volatile products in the breath that help to discriminate control subjects from patients with different pathologies or cancers. At present, we can select and analyze some previously identified volatile products, without any preliminary concentration. IMR-MS, PTR-MS and other similar techniques without any gas chromatographic separation have the advantage of less sample manipulation and require neither large collections nor high concentrations [26,27]. However, the indeterminacy of the type of the detected molecule often remains. The good sensitivity (ppb) of such equipment, which can work on-line and give results within the time of a single breath, together with the proper statistical management of multivariable data, allow for characterizing the profile of some volatile compounds and comparing different subjects.

Some slight advantages of IMR-MS over other ion attachment mass spectrometry methods seem to be related to the measurement ranges of CO_2_ in alveolar air that are wider with IMR-MS. Moreover, with such equipment, the linearity of responses is less affected by the presence of water in the exhaled breath.

In our study, we have collected and analyzed the alveolar air samples of healthy subjects and patients suffering from squamous cell lung cancer, lung adenocarcinoma and colon adenocarcinoma, using off-line IMR-MS. All cancers were histologically confirmed and defined. Our goals were: (a) to verify if typical volatile compounds (VCs) were present in each kind of cancer as compared to healthy subjects, (b) to identify patterns of volatile compounds that could identify patients with distinct cancers from controls and (c) if any volatile compound was commonly present in different kinds of cancers, as a sort of detectable biomarker in most cancer patients.

## 2. Results

In this research, 158 subjects were involved, with an average age of 67.4 years, including 70 women and 88 men. Patient characteristics are described in Table 1.

The mean age of patients with squamous lung cancer was somewhat greater than that of patients with pulmonary adenocarcinoma or adenocarcinoma of the colon. The mean age of the control subjects was 66.2 years (range 43–87), similar to that of subjects with different kinds of neoplasms (average 67.9 years; range: 43–89).

Patients operated for pulmonary neoplasia were in the following stages: in group 1 (lung adenocarcinoma) there were 36 patients (n = 19 stage I, n = 9 stage II, n = 1 stage III, n = 7 stage IV), in group 2 (lung squamous cell carcinoma) there were 25 patients (n = 6 stage I, n = 10 stage II, n = 1 stage III, n = 8 stage IV). The number of patients in different stages was too small to allow for stratified analysis by staging.

Among patients with colorectal cancer, after the histopathological examination, there were 16 patients in stage I, 15 in II and 12 in III. For nine patients, only the presence of adenomas with high-grade dysplasia was confirmed.

**Figure 1:**
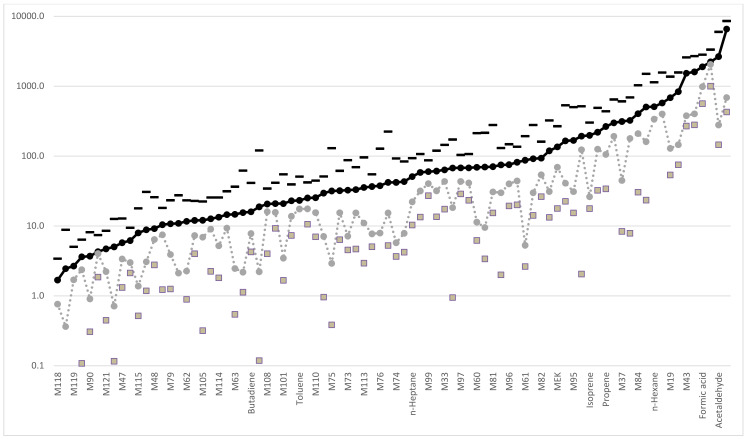
Alveolar (black dots) and environmental (gray dots) mean concentrations (expressed in ppb) of the 84 volatile organic compounds (VOCs) used for the statistical elaboration. Each dot represents the mean of 158 samples. The standard deviations are reported with a black dash for alveolar air samples and small gray squares for environmental samples. Ten compounds measured in the environment had a mean value lower than their standard deviation (such standard deviations are not included in the figure).

Figure 1 shows the mean environmental and alveolar concentration of volatile compounds measured by the IMR-MS. Environmental concentrations represent background contamination in the sites were subjects provided their alveolar air sample. The names of the 84 molecules found in higher concentrations in the alveolar air than in the environmental air are shown on the abscissa. Eleven molecules were excluded (M27, ethane, formaldehyde, methanol, ethylene, nitric oxide (NO), M31, M32, hydrogen sulfide (H2S), M46 and M49) because of their significantly higher concentration in environmental air (in a *t*-test) if compared to that in the corresponding alveolar air. We selected those compounds that were more abundant in alveolar air than in environmental air, assuming that they could give more information on the physio-pathological conditions of humans and, at the same time, reduce the interference of background contamination mainly related to environmental pollution. On the ordinate of Figure 1, the average concentrations (with its standard deviation) of the single volatile compounds expressed in ppb are reported, each point is the average of 158 alveolar or environmental values.

The iterative statistical analysis described above, by using the alveolar concentrations of the 84 molecules reported in Figure 1, with age and sex, allowed us to generate the algorithms resulting from the comparison of data between control subjects and patients with lung adenocarcinoma, lung squamous cell carcinoma or with colon adenocarcinoma.

**Figure 2:**
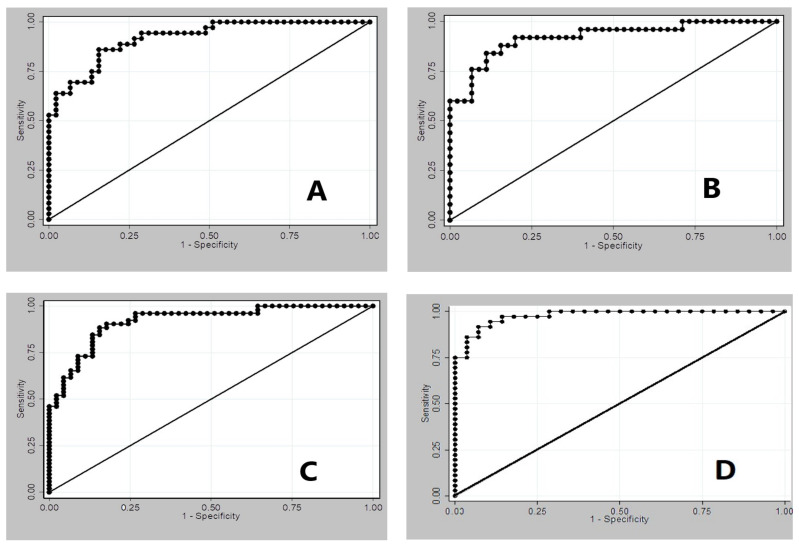
(**A**) Receiver Operating Characteristic (ROC) curve related to the final model comparing controls and patients with lung adenocarcinoma (area under the ROC = 0.9198; 95% CI 0.8637–0.9758); (**B**) ROC curve related to the final model comparing controls and patients with lung squamous cell carcinoma (area under the ROC = 0.9218; 95% CI 0.8518–0.9917); (**C**) ROC curve related to the final model comparing controls and patients with colon adenocarcinoma (area under the ROC = 0.9205; 95% CI 0.8675–0.9735); (**D**) ROC curve related to the final model comparing patients with lung squamous cell carcinoma or lung adenocarcinoma (area under the ROC = 0.9772; 95% CI 0.9499–1.0000).

To compare data from controls and patients with lung adenocarcinoma, the first least absolute shrinkage and selection operator (LASSO) logistic regression (LLR) was run with age, sex and all 84 volatile compounds. From the 50-fold cross-validation procedure, we obtained a λ-value of 4.350255, and a model with 11 volatile compounds plus age; all other variables (73 compounds and sex) had regression coefficients equal to zero and were thus discarded. The second LLR (LASSO followed by an adaptive version of LASSO) confirmed a model including age and the 11 volatile compounds (Table 2). This final model has an area under the Receiver Operating Characteristic (ROC) Curve (AUC) curve of 91.98% (Figure 2A); the performance of the model for sensitivity levels above 86% are reported in Table 3.

Table 4 reports the main statistical parameters of the concentrations in alveolar air (expressed in ppb) of the volatile compounds selected by the final model, both for patients with lung adenocarcinoma and controls. Acetic acid, ammonia, acetaldehyde and pentane were the known and calibrated molecules included in the algorithm, together with six other molecules known only by their molecular weight.

The same statistical approach was used for comparing data from control subjects and patients with lung squamous cell carcinoma. The following results were obtained: the second LLR selected age, sex and nine VCs (reported in Table 5) for the final model, which has an AUC of 92.18% (Figure 2B). In Table 6, the performance of the model for sensitivity levels above 87% is reported.

Table 7 reports the main statistical parameters of the nine compound concentrations in alveolar air (expressed in ppb) identified by the final model, both for patients with lung squamous cell carcinoma and controls.

Comparing data obtained from control subjects and patients with lung cancer (lung adenocarcinoma or squamous cell carcinoma), we obtained an algorithm including sex and 13 volatile compounds. These results were less promising than the ones found by the algorithms obtained for the two single kinds of lung cancers, as shown in Table 8, where the performance of the model for sensitivity levels above 83% are included. We tested the equality of the ROC areas with the Stata command roccomp, based on DeLong et al. [28], comparing the results of the lung cancer generic model first with lung adenocarcinoma and then with squamous cell lung carcinoma, respectively, on controls vs. lung adenocarcinoma, and controls vs. squamous cell lung carcinoma. In both cases, the areas were larger in the specific models, but the difference was not significant, even if it was more evident for the squamous cell lung carcinoma (comparison of generic lung model with lung adenocarcinoma model: ROC areas 0.9136 vs. 0.9198, *p*-value = 0.7844; comparison between generic lung model and squamous cell carcinoma: ROC areas 0.8773 vs. 0.9218, *p*-value = 0.1770).

Despite the relatively small number of cases, we then tried to separate lung adenocarcinoma from lung squamous cell carcinoma, excluding controls. The final model comprised age and sex, plus 12 VCs (Table 9) with an AUC of 0.9772 (Figure 2D). In Table 10, the performance of the model for sensitivity levels for the identification of lung adenocarcinoma is reported, while specificity for lung adenocarcinoma can be read as sensitivity for lung squamous cell carcinoma, and vice versa.

**Figure 3:**
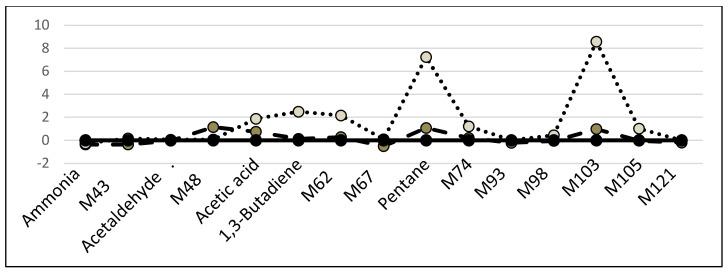
Profiles of the volatile compounds in patients with lung adenocarcinoma (gray circles and dotted line) or lung squamous cell carcinoma (dark gray circles and dashed line) standardized to mean and standard deviation of controls (black circles and continuous line).

The profiles of volatile compounds found in the two types of lung cancers were different, as reported in Figure 3: five volatile compounds (acetic acid, ammonia, M43, acetaldehyde and M48) were modified in both situations, but 10 other volatile compounds had a different behavior, suggesting dissimilar biological–biochemical conditions.

The comparison of data obtained from control subjects and patients with colon adenocarcinoma by using the predictive model on a LASSO logistic regression allowed us to obtain an algorithm which included sex, age and 13 VCs, reported in Table 11. This model has an AUC of 92.05% (Figure 2C); the performance of the model for sensitivity levels above 88% are reported in Table 12.

**Figure 4:**
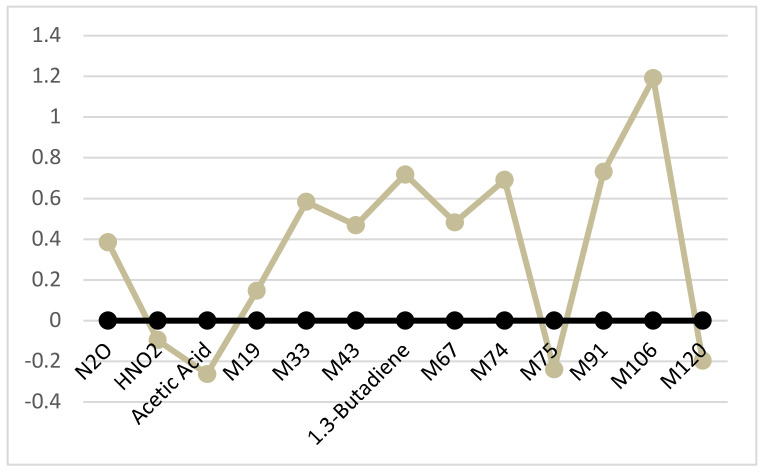
Profile of the volatile compounds of patients with colon adenocarcinoma (gray circles and line) standardized to mean and standard deviation of controls (black circles and line).

Table 13 reports the main statistical parameters of alveolar concentrations of thirteen VCs selected by the final model both for patients with colon adenocarcinoma and controls. Such parameters are depicted in Figure 4, which points out that the profiles of volatile compounds suggested by the model are different in control subjects when compared to those found in patients with colon adenocarcinoma.

Nitrogen compounds such HNO_2_ and N_2_O identified by the algorithm for discriminating patients with colon cancer from control subjects present an average concentration in alveolar air four to eight times higher than in environmental air; such environmental pollution is too low and cannot interfere with the significance of biological sources of these nitrogen compounds. These obligatory inorganic metabolites of denitrifying bacteria [29] were never identified in breath with the GC-MS method, but often studied in the breath condensate [30].

## 3. Discussion

With this study, we show that alveolar air analysis, using IMR-MS, can discriminate control subjects from patients with lung cancer (adenocarcinoma or squamous cell carcinoma) or colon cancer. The profiles of volatile compounds selected by the calculated algorithms, using the statistical analysis LASSO, allow us to discriminate different groups of patients with good sensitivity and specificity. We collected individual alveolar air samples in small, airtight glass containers which were subsequently analyzed by IMR-MS. The same analysis can also be performed on the spot, directly from a subject (patient or control) and the results can be available in less than 30 s. These can be compared to the profiles of volatile products previously obtained from selected groups of cases and controls.

Studying biomolecules present in the breath has raised particular interest in terms of detecting some biological markers related to specific pathologies. Recent studies do not indicate the presence of “new compounds” which are specific to pathological alterations. Miekisch et al. [31] noted that, to date, no compound has been identified in the breath of patients but not in healthy controls. These findings were also confirmed by the literature concerning lung cancers for which many volatile products have been identified using CG-MS methods. In spite of these “gold standard” analyses, different sampling and preconcentration concentration methods of VOCs in breath and dissimilar separation techniques reveal different substances, and only a few products were confirmed by different research groups.

The literature of the last 10 years identified the compounds reported in Table 14 as possible biomarkers of lung cancer. Reliable methods, such as GC-MS, were used in all these studies, but only 1-butanol was found by two research groups. The other suggested biomarkers were identified by only one working group.

Saalberg and Wolff [37], in a comprehensive review about VOC breath biomarkers in lung cancer from 1985 to 2015, found that the number of the most frequently emerging biomarkers, identified by four or five research groups, was only six (2-butanone, 1-propanol, isoprene, ethylbenzene, styrene and hexanal). Another nine and 15 compounds were, respectively, detected by three and two different groups of researchers. The number of biomarkers identified by only one working group was 43. It is not easy to understand the reason for such differences, even if the analytical types of equipment (usually GC-MS) used represented the gold standard for these kinds of analysis. The low concentrations of VOCs in exhaled breath, often lower than the quantification limits, the high water content of breath, which complicates the quantification of trace VOCs, and the background VOC content of the ambient air are problems that make these studies difficult, and do not facilitate the comparison between the results obtained by different working groups.

A critical aspect of some of these studies relates to the proposal to use products typically associated with environmental pollution as lung cancer biomarkers. It is difficult to think that styrene, cyclohexane, xylene or ethylbenzene could give information about the biological modifications related to cancerogenic processes. Their presence in human bodies must be attributed to the ubiquitous pollution which favors their uptake and their trend to be accumulated in fat tissues. Such tissues release these compounds very slowly, through the breath, only when environmental pollution is very low. From the medical point of view, they can be hardly related to cellular physiology or pathology even if slight metabolic differences could be conjectured in patients compared to controls. This condition is very difficult to demonstrate and, in our opinion, the probability that alveolar concentrations of such compounds could be related to biological processes is very low. On these bases, we preferred to include in our statistical processes only compounds with alveolar concentrations higher than the environmental ones.

The algorithms obtained allowed us to identify some volatile compounds (n-pentane, 1,3-butadiene, acetic acid, acetaldehyde, ammonia and dinitrogen oxide) previously reported as relevant in exhaled air. Both n-pentane and 1,3-butadiene were previously recognized discriminants of lung cancer [11,38,39,40,41,42], mainly related to lipid peroxidation.

Acetic acid, together with limonene, decanoic acid and furfural, were the volatile compounds with the highest capacity to discriminate tissues with breast cancer from tissues that were cancer free [43]. The presence of acetic acid in exhaled air was confirmed in both controls and in patients suffering from gastro-esophageal reflux disease or cystic fibrosis [44,45]. Our results on acetaldehyde, which was lower in the alveolar air provided by patients with lung adenocarcinoma, are in line with the in vitro results published by Sponring et al. [46].

Previously, breath ammonia was measured in healthy volunteers and in patients with chronic kidney disease by using an electrochemical sensor [47]. Elevated blood ammonia levels (and likely in alveolar air) are associated with a variety of pathological conditions, such as liver and kidney dysfunction, Reye’s syndrome and several inborn errors of metabolism [48]. The breath ammonia concentration is lower in cancer patients than in controls; such lower concentrations were also found in inflammatory bowel diseases [49].

In our research, some volatile compounds useful for cancer discrimination were identified exclusively by their molecular weight. Previous research works help us to make some assumptions. Bajtarevic et al. [41] suggested that M43 could be ethylenimine, which was also found in patients with lung cancer. Based on the molecular weight, we can assume that M48 could be methanethiol [50] and M62 and M76 could be, respectively, dimethyl sulfide and carbon disulfide [35]. M74 could be 1-Hydroxy-2-propanone [35], M98 could be methylcyclohexane [14] and M121 could be ethylaniline [9]. The reported research works confirmed the presence in exhaled breath of such compounds by using GC-MS methods both in lung cancer patients and in control subjects. Future laboratory experiments will verify these hypotheses; by using the IMR-MS, we have to identify the best analytical conditions for the just reported compounds. Such conditions are not the same for other products with the same molecular weight despite slight possible interferences.

Our results, highlighted in Figure 2 and in Table 9 and Table 10, suggest that patients with lung adenocarcinoma and those with lung squamous cell carcinoma have different alveolar profiles of volatile compounds. The two profiles, reported in Figure 2, are standardized to the mean and standard deviation of the controls. Some products (acetic acid, ammonia, acetaldehyde, M43, M103) were selected by algorithms as discriminants of lung cancer, but other molecules show concentrations which differentiate the two kinds of lung cancer. In the specific literature about breath analysis, Peled et al. [51] were able to differentiate between adenocarcinoma and squamous cell lung cancer in patients by using a chemical nanoarray containing gold nanoparticle sensors. In vitro experiments also showed that different kinds of lung cancer cell lines release or consume various volatile molecules, suggesting a specific metabolic pattern among diverse lung cancer cells [46,52].

Our data confirm that the appearance of colon cancer is accompanied by several biological modifications and changes in the concentration of volatile physiological products in the breath of patients. Dinitrogen oxide, nitrous acid, acetic acid and 1,3-butadiene were the volatile products that our algorithm recognized as discriminants for this pathological condition. Different processes of denitrifying and nitrifying related to the intestinal microbiome could explain the changes in alveolar concentration of both dinitrogen oxide and nitrous acid in patients with colon adenocarcinoma compared to controls.

Alveolar 1,3-Butadiene concentrations were modified both in patients with lung squamous cell carcinoma and colon carcinoma. This product was predominantly measured in the breath of smokers but was also present in non-smoker subjects [53,54]; at present, it is difficult to theorize a physio-pathological source. Among the volatile products selected by the algorithm and identified exclusively by the molecular weight, we can hypothesize that M106 could be xylene (1,3- and 1,4-dimethylbenzene), which was also found in the breath of patients with colon cancer by Altomare et al. [55] and Di Lena et al. [56].

Leja et al. [57] assessed the effects of some conditions which change the gut microbiome based on the breath test results. They confirmed that Helicobacter pylori eradication therapy, as well as bowel cleansing before colonoscopy, can modify the breath profile: only three among 133 studied VOCs were identified as significantly increased (α-pinene, ethyl acetate and acetone).

In two subsequent papers, Altomare et al. [14,55] found that nonanal, 4-methyl-2-pentanone, decanal, 2-methylbutane, 1,2-pentadiene, 3-methylpentane, methylcyclopentane, cyclohexane, methylcyclohexane, 1,3-dimethylbenzene and 1,4-dimethylbenzene were discriminants between healthy subjects and patients with colon adenocarcinoma.

Wang et al. [9] studied the VOCs in the exhalations of patients with colorectal cancer. Their results showed that in the cancer group, eight metabolic biomarkers were significantly more expressed in the group of colorectal cancer patients than in the controls. Amal et al. [58] collected 418 breath samples from 65 patients with colorectal cancer, 22 with advanced or non-advanced adenomas and 122 control cases. Their results revealed four significant VOCs that identified the tested groups: these were acetone and ethyl acetate (higher in colorectal cancer group) and ethanol and 4-methyl octane (lower in colorectal cancer group). Di Lena et al. [55] carried out a review on VOC biomarkers for colorectal cancer. Only two VOCs in breath (1,3-dimethylbenzene and 4-methyloctane) were found by more than one group of researchers. Arasaradnam et al. [59] identified some VOCs in the urine that allowed them to discriminate with good sensitivity and specificity control subjects from patients with colon neoplasms. It should be noted that VOCs from the feces can also indicate the presence of intestinal inflammatory processes or even colon neoplasms [60].

In our study, among the volatile compounds able to discriminate the three different kinds of cancers, only acetic acid and M43 (which could be ethylenimine, which has a molecular weight of 43 Da and was found by Bajtarevic et al. [41] in patients with lung cancer) were identified by the algorithms as products modified in the alveolar air of all patients with cancer. Acetic acid presented a high contribution towards the discrimination of breast cancer and cancer-free tissues [43].

The different profiles of volatile compounds we found in patients with cancer and the revised literature on this issue [37,61] suggest that each cancer, coming from specific cells, should be associated with typical variations of the breath profile that are significantly different from subjects without any cancer. In different stages of cancer, the VOC profile in breath changes in the sense that some volatile compounds are increased while others are reduced; some of them start to modify along the multistep process of cancer and the intensity of these changes could identify different stages of cancer.

Some weaknesses and strengths of this research are listed below. The number of subjects used for each population is small if compared to other studies in this field. Moreover, the discrimination of cancer patients from control subjects was performed by using some unidentified substances, rendering the knowledge about the physicochemical process related to the concentration of volatile compounds in exhaled air fairly limited. The equipment we used does not always allow the identification of all compounds and this is a weakness. However, it can work without any previous preconcentration of samples and can give results within the time of a single breath when working on-line; these are important strengths.

Other important strengths of this study are the statistical models we used. These models allow us to calculate algorithms able to discriminate patients from control subjects with high probabilities, even in the presence of relatively small samples and of a high number of compounds to be considered.

After identifying the algorithms, the analysis of some volatile products in alveolar air by using on-line IMR-MS or similar analytical tools overcomes the differences in the methods of collection and concentrations of individual samples and favors speed, security, minimal invasiveness and low costs for new promising breath studies.

## 4. Materials and Methods

### 4.1. Study Population

The study population, recruited between 2012 and 2015, included the following four groups: (1) patients with lung adenocarcinoma, (2) patients with lung squamous cell carcinoma, (3) patients with colorectal cancer, (4) a control group of healthy subjects.

The study was approved by the Ethics Committee of the Careggi University Hospital (Rif. n. 27/12) and was conducted according to the Helsinki Convention. All involved subjects provided written informed consent for their participation in the research and their willingness to cooperate with the breath collecting procedure.

All checked patients provided their alveolar samples in the morning. At sampling, all enrolled patients were fasting, to avoid the possible effect of food or its metabolites on the profile of volatile compounds. All hospitalized controls were sampled in the morning and had been fasting at least since midnight. Outpatient controls had been fasting for at least two hours before sampling. They were required not to smoke and drink alcohol from midnight on.

#### 4.1.1. Patients with Lung Cancer (Group 1 and 2)

Patients with lung cancer, at different stages, were enrolled at the Department of Experimental and Clinical Medicine of the Careggi University Hospital. Each patient, after routine examinations, underwent a complete diagnostic workup with chest computed tomography (CT), 18-F-fluorodeoxyglucose positron emission tomography (18-F-FDG-PET-TC) and histological confirmation. No medical treatments (chemotherapy/radiotherapy) were performed on the patients before alveolar air sampling.

Exclusion criteria were: a second primary lung cancer (synchronous or metachronous), and other malignant diseases during the previous five years before enrolment. Patients with lung cancer were planned for surgical treatment, and alveolar air samples were collected 1–3 days before surgery, to avoid any interference with the volatile compounds of the drugs used in the operating room or the stress related to the surgical procedure. The patients’ tumors were staged according to the cancer staging manual of the American Joint Committee on Cancer [62] used at the time they had surgery.

#### 4.1.2. Patients with Colorectal Cancer (Group 3)

Patients with colorectal cancer and those with endoscopically unresectable adenomas with areas of different degrees of epithelial dysplasia were enrolled at the Colorectal Surgical Unit of the Department of Experimental and Clinical Medicine at the Careggi University Hospital. The diagnosis was determined through pancolonoscopy with multiple biopsies. In case of an incomplete colonoscopy, a CT colonography was performed. The pre-treatment tumor stage was determined in all patients by a chest and abdominal CT scan. Exclusion criteria were the presence of metastatic disease of colon cancer, other malignant diseases during the previous five years before enrolment, previous treatment with chemo-radiotherapy and any ongoing respiratory disease such as chronic obstructive pulmonary disease (COPD).

The presence of adenomas with high-grade dysplasia was considered as colorectal cancer.

All enrolled patients underwent curative standard colectomy and en bloc regional lymphadenectomy. All surgical procedures were performed either via conventional open or laparoscopic access. The alveolar air samples were collected the day before surgery. Tumors were staged according to the American Joint Commission on Cancer/International Union Against Cancer TNM staging system [63].

#### 4.1.3. Control Subjects

It is difficult to identify “perfectly healthy control subjects” with the same age as patients with lung or colon neoplasm. We selected people hospitalized and undergoing clinical tests that did not detect any neoplasm or other important diseases. Several of our control subjects were screened for lung cancer with a computed axial tomography scan because of their previous risk from smoking. Other control subjects were hospitalized patients planned for slight surgical interventions, such as venous or hemorrhoidal varices, inguinal hernias, etc. Such patients were checked with a standard chest X-ray (which was negative for lung diseases) and several other biochemical examinations which denied liver cirrhosis and any immunological or inflammatory diseases.

Other control subjects were outpatients selected among people periodically checked for their working conditions or slight respiratory disorders (e.g., non-allergic rhinitis, mild bronchial asthma) hypertension or psychological disorders, but in good general health conditions. Moreover, the same pathologies were also recorded in several patients with cancer. Exclusion criteria for control subjects were the presence of acute respiratory tract infections, previously diagnosed neoplasms or significant pathologies of the central nervous system. Control subjects were chosen to have ages comparable with patients.

### 4.2. Alveolar Air Sampling

For exhaled breath sampling, subjects were asked to make one deep exhalation inside a hand-device called a Bio-VOC breath sampler^®^ (Markes International Ltd. Rhondda Cynon Taff. UK), which is a special 250 mL air syringe able to avoid any re-breath phase, as previously described [17,21]. Through the syringe, the breathed air flows into a 20 mL glass vial with a wide-bordered opening, formerly sterilized and kept at 80 °C for at least 24 h to avoid the presence of environmental pollutants. After completing exhalation, the glass vial (containing the last part of the exhaled air, which is the alveolar air) was crimped airtight using a Teflon septum (PTFE) and an aluminum ring. Two samples of expired air were collected for each subject. In the same time and in the same room, a sample of environmental air was also collected into a 20 mL sterilized vial and treated as alveolar samples.

The sample tubes were then kept at −20 °C until analysis. The concentration of CO_2_ registered at the analysis was used to assess the quality of the samples: alveolar air samples with CO_2_ levels lower than 2% were discarded and excluded from the statistical analysis, because these measurements suggested these vials had either not been crimped airtight or the alveolar air sampling had not been correctly performed.

### 4.3. Equipment

An AirSense Compact analyzer and a V&F autosampler (V&F Analyse- und Messtechnik GmbH. Absam, Austria) were used for analyzing the volatile compounds present in the samples, as previously described [17,64,65]. The AirSense Compact analyzer consists of a conventional electron impact MS and a highly sensitive ion molecule reaction mass spectrometer (corresponding to chemical ionization mass spectrometry). The first one was used for the analysis of carbon dioxide and oxygen present in alveolar or environmental air, while the second one, with a soft ionization unit (that qualifies as fast atom bombardment) analysed other volatile compounds present in the samples.

The ionization process for the detection of sample molecules was performed via ion beams interacting with the gas sample. Mercury or xenon were first ionized by electron impact. These primary molecule ions then produced a smooth charge exchange with the breath sample molecules. This procedure is termed ion molecule reaction (IMR). After this soft ionization, the breath ions were separated in a quadrupole mass filter that allowed the subsequent quantification of the single compounds. The vials from checked subjects were placed in the V&F autosampler, heated up to 65 °C for one hour and dynamically transferred to the V&F AirSense Compact. In a few seconds, the concentration of 95 volatile compounds (with masses between 16 and 123) present in the air samples can be obtained. These products mainly represent molecules existing in traces in the sample but may, in some cases, also represent fragments of other molecules generated by the soft ionization occurring in the instrument.

Of these 95 volatile compounds, twenty-eight had a known chemical structure (directly or indirectly calibrated), while 67 products were known only by their molecular weight.

The following sixteen products were directly calibrated: formaldehyde, acetonitrile (ACN), formic acid, acetic acid, acetaldehyde, methyl ethyl ketone (MEK), isoprene, acetone, methanol, n-propanol, n-butanol, n-pentane, n-hexane, benzene, toluene and n-heptane. A mixture of such compounds (liquids) using more for those usually present in higher concentrations in alveolar air (acetone) was prepared. Five microliters of this mixture were put in a hot bottle of 2750 mL in volume which had been cleaned with pure helium and tightly closed. The bottle was put over a hot magnetic stirrer. After some minutes, 100, 200, 400 or 800 μL were transferred with a hot syringe for gas into glass vials of 20 mL; the obtained concentrations were in the range of 100–1000 ppb (apart from acetone which was about 3000–10,000 ppb). The obtained results were used to calculate the concentrations in our samples collected in 20 mL vials.

Ten other volatile compounds (methane, acetylene, ethane, ethylene, ammonia (NH_3_), propene, 1,3-butadiene, nitrous acid (HNO_2_), nitric oxide (NO) and dinitrogen oxide (N_2_O)) were calibrated to the sensitivity of one directly calibrated component (benzene). Their calibration coefficients were calculated by connecting the AirSense analyzer to cylinders with a known concentration of each gas. Other volatile compounds, named as “M” followed by the molecular weight of the detected compounds, were also calibrated on the sensitivity of benzene. This kind of semi-quantitative calibration procedure is commonly used in multicomponent analytical devices. We use the words “volatile compounds” (not VOCs) because some of the measured compounds, such as ammonia, nitrous acid and others, are not considered organic compounds. The calibration of the mass spectrometer was also performed by using calibration mixtures containing CO_2_ and O_2_ at 10% and 5%, respectively (from Messer Italia spa. Settimo Torinese, Italy). The measured gas compounds are given as absolute concentrations (ppb) and volume percent for CO_2_ and O_2_.

The variation coefficients in volatile compound measurements by the AirSense were reported elsewhere [21] and were lower than 20%, except for acetone (24%) and acetic acid (35%). The percentage of carbonic anhydride was tested to confirm the alveolar origin of the collected air (CO_2_ > 2%). The reliability and validity of measurements are reported in a previous paper; our environmental and alveolar samples give results in the ranges already reported in the international literature [17].

We did not use the on-line condition because the alveolar air samples were collected over a period of 3 years and in different hospitals; the equipment, therefore, could not have been constantly moved.

### 4.4. Statistics

Taking into account the large number of independent variables involved in the analysis, we decided to adopt a least absolute shrinkage and selection operator (LASSO) logistic regression (LLR) for the elaboration of the predictive models [17,21,66,67]. The LASSO is a penalized estimation method which avoids overfitting caused by collinearity or high dimensionality of independent variables through the shrinking of the estimated regression coefficients. A tuning parameter λ controls the amount of shrinkage applied to the estimates. The shrinkage of some coefficients to zero reduces the number of covariates in the final model.

As suggested by Huang et al. [67], and as tested in previous studies [17,21], we used an iterated LLR approach. Huang et al. [67] demonstrated the consistency of the LASSO and the oracle property of the iterated LLR in sparse, high-dimensional settings. Briefly, we first used an LLR to reduce the number of variables involved in the model, eliminating all variables if coefficients were 0 and ignoring their coefficients if these were >0. We then included the remaining variables in a two-step iterated LLR [67]: a first LLR to generate penalized weights to be used in an adaptive LLR, as described by Huang et al. [67]. Penalized weights were calculated as inverse logistic regression coefficients. For this last regression, confidence intervals of the regression coefficients were calculated with a 10-fold iterated bootstrap procedure. A 50-fold cross-validation was applied to all steps of the LLR, and independent variables (molecules) were standardized to allow optimal penalization. As proven in previous studies [17,21], the variable reduction approach, applied with the first LASSO, allowed us to obtain better performing final models, in terms of sensitivity and specificity.

All analyses were carried out with Stata/IC 14.2 (StataCorp LP. College Station, TX, USA) and R 3.5.3 (The R Foundation for Statistical Computing. Vienna, Austria) and the “penalised” [68] and “polywog” packages (Kenkel B. Signorino CS. polywog: Bootstrapped Basis Regression with Oracle Model Selection, version 0.2-0. 2012).

## 5. Conclusions

Our results emphasize the differences among the profiles of volatile compounds present in the alveolar air of patients with different kinds of cancer in the same tissue, such as the lungs (lung adenocarcinoma and lung squamous cell carcinoma). Some biomarkers (acetic acid, ammonia, acetaldehyde, M43, M103) have a similar behavior in the alveolar air, but others show concentrations which differentiate the two kinds of cancers. Additionally, the results obtained from patients with colon adenocarcinoma suggest that each kind of cancer, arising from different cells, has a specific profile of alveolar volatile compounds related to biochemical processes, particular to each kind of cell. When a cell becomes a cancer cell, some of its biochemical reactions are modified and a new pathological condition begins which changes the amount of some of the volatile products synthesized by the same cells, with an alteration of the alveolar profile of volatile compounds. Among the measured compounds in alveolar air, only acetic acid was identified by the algorithms as a biomarker of the three different kinds of cancer we studied. The availability of the algorithms we calculated and recent analytical tools, such as IMR-MS or PTR-MS or similar equipment, which can provide on-line information of the alveolar profile of numerous volatile products, gives new thrusts to diagnostics and physio-pathological studies of different kinds of cancers and other diseases. The on-line working condition gives results within the time of a single breath and overcomes the differences in the method of collection and the concentration of individual samples favoring speed, security, minimal invasiveness and low costs for new promising breath studies.

## Figures and Tables

**Table 1:** Characteristics of subjects enrolled in the study: age and smoking status.

	Control Subjects * n = 45 (men = 30)	Patients with Lung Adenocarcinoma n = 36 (men = 21)	Patients with Lung Squamous Cell Carcinoma n = 25 (men = 13)	Patients with Colorectal Cancer n = 52 (men = 24)
**Age**				
Men	64.5 (43–87)	69.0 (45–80)	72.5 (55–80)	66.5 (43–81)
Women	68.0 (44–83)	62.0 (52–85)	72.5 (65–86)	68.5 (44–89)
**Smoking status**				
Non-smokers	28 (62.2%)	3 (8.3%)	0	42 (80.8%)
Former smokers	14 (31.1%)	5 (13.9%)	3 (12%)	6 (11.5)
Current smokers	3 (6.7%)	28 (77.8%)	22 (88%)	4 (7.7)
Pack-years	0 (0–90)	10 (0–30)	20 (10–30)	0 (0–80)

* Values are number of observations or median and range (in brackets).

**Table 2:** Lung adenocarcinoma model obtained from the first least absolute shrinkage and selection operator (LASSO) logistic regression: regression coefficients of the second LASSO logistic regression (LLR) with 95% confidence intervals.

Variables	Coefficients	95% CI *
Intercept	+0.0307786	+0.768–+8.541
Age	−0.0002355	−0.134–+0.000
Acetic Acid	+0.0018644	+0.000–+0.004
Ammonia (NH_3_)	−0.0007868	−0.004–+0.000
M43	−0.0009794	−0.005–+0.002
Acetaldehyde	−0.0003237	−0.001–+0.000
M48	+0.0179830	+0.001–+0.384
M62	+0.0851835	−0.044–+0.346
M67	−0.0833412	−0.272–−0.032
Pentane	+0.0285685	+0.000–+0.080
M93	−0.0418881	−0.183–+0.058
M98	−0.0065165	−0.020–+0.057
M103	+0.0161747	−0.312–+0.044

* CI: confidence interval.

**Table 3:** Performance of model comparing patients with lung adenocarcinoma (ADK lung) to controls (in parentheses, the number of correctly classified cases).

Predicted Probability	Sensitivity in ADK Lung Cases (n = 36)	Specificity in Overall Controls (n = 45)
0.1730204	100.00% (36)	48.89% (22)
0.1791286	97.22% (35)	51.11% (23)
0.2633279	94.44% (34)	71.11% (32)
0.3165469	91.67% (33)	73.33% (33)
0.3633479	88.89% (32)	77.78% (35)
0.3929707	86.11% (31)	84.44% (38)

**Table 4:** Main statistical parameters related to volatile compounds selected by the final predictive model to discriminate between control subjects and patients with lung adenocarcinoma (data in ppb).

	Acetic Acid	Ammonia (NH3)	M43	Acetaldehyde	M48	M62	M67	Pentane	M93	M98	M103
Control subjects											
Median	1124.0	160.6	1193.0	818.0	6.8	8.7	34.4	64.3	31.9	58.4	4.2
Arithmetic mean	1336.8	602.3	1430.5	1976.1	7.8	9.2	35.9	64.5	35.7	67.1	7.1
Range	402.5–3742.7	52.1–6375.2	606.5–6938.6	202.2–9423.5	3.0–17.3	2.7–19.9	12.5–75.5	29.5–130.2	6.3–299.6	23.2–256.9	1.3–31.7
Patients with lung adenocarcinoma											
Median	1872.8	173.6	927.9	580.2	6.3	8.9	27.2	78.8	24.2	62.6	6.1
Arithmetic mean	1855.5	171.1	1071.5	1901.1	11.6	10.5	28.6	85.6	26.9	65.0	14.4
Range	376.1–3999.2	32.6–384.4	284.5–2773.8	105.5–11,089.2	1.6–199.3	1.9–31.1	4.0–52.1	37.4–188.0	4.4–92.5	26.3–135.4	0.6–131.6

**Table 5:** Lung squamous cell carcinoma model obtained from the first LASSO logistic regression: regression coefficients of the second LLR with a confidence interval between 2.5% and 97.5%.

Variables	Coefficients	95% CI *
Intercept	−6.8203051	−8.816–−0.266
Sex	2.0805581	+0.311–+2.933
Age	0.0489415	+0.000–+0.075
Acetic Acid	0.0009828	+0.000–+0.002
Ammonia (NH_3_)	−0.0004396	−0.001–+0.000
M43	−0.0003079	−0.001–+0.000
Acetaldehyde	−0.0001551	−0.0005–+0.000
M48	−0.0513673	−0.357–+0.000
1,3-Butadiene	0.0215291	−0.116–+0.038
M74	0.0247382	+0.000–+0.065
M105	0.1281680	−0.118–+0.153
M121	−0.2698088	−0.247–+0.000

* CI: confidence interval.

**Table 6:** Performance of model comparing patients with lung squamous cell carcinoma (KSQ lung) to controls (in parentheses, the number of correctly classified cases).

Predicted Probability	Sensitivity in KSQ Lung Cases (n = 25)	Specificity in Overall Controls (n = 45)
0.0427018	100.00% (25)	28.89% (13)
0.1533398	96.00% (24)	60.00% (27)
0.3268180	92.00% (23)	80.00% (36)
0.3502457	88.00% (22)	84.44% (38)

**Table 7:** Main statistical parameters related to the 9 volatile compounds selected by the final predictive model to discriminate between control subjects and patients with lung squamous cell carcinoma (data in ppb).

	Acetic Acid	Ammonia (NH3)	M43	Acetaldehyde	M48	1.3-Butadiene	M74	M105	M121
Control subjects									
Median	1124.0	160.6	1193.0	818.0	6.8	10.9	23.4	9.4	3.7
Arithmetic mean	1336.8	602.3	1430.5	1976.1	7.8	11.8	31.6	9.9	5.1
Range	402.5–3742.7	52.1–6375.2	606.5–6938.6	202.2–9423.5	3.0–17.3	3.2–25.6	9.7–90.7	3.7–25.9	1.1–23.5
Patients with lung squamous cell carcinoma									
Median	2119.7	219.9	1260.8	1362.2	6.4	13.6	37.8	11.1	4.5
Arithmetic mean	2627.4	276.2	1599.1	2120.2	8.1	27.6	58.9	15.2	5.1
Range	478.9–8398.6	42.7–947.4	350.6–7699.2	116.7–9456.8	2.0–26.8	3.2–255.1	9.8–231.8	5.3–57.3	1.5–10.2

**Table 8:** Performance of model comparing patients with lung cancer (lung adenocarcinoma and squamous cell carcinoma) to controls (in parentheses, the number of correctly classified cases).

Predicted Probability	Sensitivity in Lung Cancer Cases (n = 61)	Specificity in Overall Controls (n = 45)
0.2023985	100.00% (61)	33.33% (15)
0.2269116	98.36% (60)	40.00% (18)
0.3327646	96.72% (59)	60.00% (27)
0.3458832	95.08% (58)	62.22% (28)
0.3995484	93.44% (57)	64.44% (29)
0.4249765	90.16% (55)	68.89% (31)
0.4429406	88.52% (54)	73.33% (33)
0.4653800	86.89% (53)	75.56% (34)
0.4811265	85.25% (52)	77.78% (35)
0.4998874	83.61% (51)	80.00% (36)

**Table 9:** Lung adenocarcinoma vs. squamous cell carcinoma model obtained from the first LASSO logistic regression: regression coefficients of the second LLR with 95% confidence intervals.

Variables	Coefficients	95% C.I. *
Intercept	+22.1958604	+11.232–+32.608
Sex	−3.3337238	−5.149–−0.257
Age	−0.1922859	−0.328–−0.024
Methane	+0.3596812	+0.000–+1.162
Acetic Acid	−0.0012951	−0.003–+0.000
Ammonia	−0.0058067	−0.008–+0.000
Acetaldehyde	+0.0002347	0.000–+0.000
M47	+0.2469311	+0.000–+0.760
1,3-Butadiene	−0.0163303	−0.105–+0.000
M67	−0.0061998	−0.086–+0.000
M74	−0.0294491	−0.059–+0.000
M99	−0.0591310	−0.187–+0.000
M109	−0.1435140	−0.306–+0.000
M120	+0.8725725	+0.000–+1.436
M123	−0.0316772	−0.129–+0.000

* C.I.: confidence interval.

**Table 10:** Performance of model comparing patients with lung adenocarcinoma to patients with squamous cell carcinoma (in parentheses, the number of correctly classified cases).

Predicted Probability	Sensitivity in Lung Adenocarcinoma Cases (n = 36)	Sensitivity in Squamous Cell Carcinoma Cases (n = 28)
0.2556554	100.00% (36)	71.43% (20)
0.4313545	97.22% (35)	85.71% (24)
0.5177129	94.44% (34)	89.29% (25)
0.5752254	91.67% (33)	92.86% (26)
0.5956354	88.89% (32)	96.43% (27)
0.7448977	75.00% (27)	100.00% (28)

**Table 11:** Colon adenocarcinoma model obtained from the LASSO logistic regression: regression coefficients of the second LLR with a confidence interval between 2.5% and 97.5%.

Variables	Coefficients	95% CI *
Intercept	−2.737	−8.821–+6.131
Sex	+0.8569	+0.000–+1.952
Age	+0.008	−7.881 × 10^−4^–+0.084
Dinitrogen Oxide (N_2_O)	+8.700 × 10^−5^	−4.721 × 10^−4^–+0.002
Nitrous Acid (HNO_2_)	−0.026	−1.029–+0.000
Acetic Acid	−5.426 × 10^−4^	−0.027–+0.000
M19	−0.002	−4.165 × 10^−3^–+0.000
M33	+0.009	+0.000–+0.483
M43	+3.467 × 10^−4^	+0.000–+0.002
1,3-Butadiene	+0.004	−0.228–+1.387
M67	+0.029	+0.000–+0.049
M74	+0.0172	+1.751 × 10^−3^–+1.289
M75	−0.0833	−2.934–−0.015
M91	+0.103	+0.000–+0.159
M106	+0.027	+0.000–+1.545
M120	−0.0917	−7.893–+0.000

* CI: confidence interval.

**Table 12:** Performance of model comparing patients with colon adenocarcinoma to controls (in parentheses, the number of correctly classified cases).

Predicted Probability	Sensitivity in Colon Adenocarcinoma Cases (n = 52)	Specificity in Overall Controls (n = 45)
0.2321	100.00% (52)	35.56% (16)
0.3786	96.15% (50)	73.33% (33)
0.4125	92.31% (48)	75.56% (34)
0.4824	90.38% (47)	82.22% (37)
0.4988	88.46% (46)	84.44% (38)

**Table 13:** Main statistical parameters related to volatile compounds selected by the final predictive model to discriminate between control subjects and patients with colon adenocarcinoma (data in ppb).

	N_2_O	HNO_2_	Acetic Acid	M19	M33	M43	1.3-Butadiene	M67	M74	M75	M91	M106	M120
Control subjects													
Median	6438.0	21.3	1124.0	350.4	46.1	1193.0	10.9	34.4	23.4	6.7	6.7	9.4	3.2
Arithmetic mean	6311.9	25.6	1336.8	623.2	51.8	1430.5	11.8	35.9	31.6	13.5	9.0	11.2	3.9
Range	2323.5–11,934.3	10.1–70.7	402.5–3742.7	84.3–1953.8	8.0–200.6	606.5–6938.6	3.2–25.6	12.5–75.5	9.7–90.7	1.7–101.4	2.2–35.3	3.9–37.6	0.6–19.4
Patients with Colon Adenocarcinoma													
Median	7306.1	23.2	1015.1	590.0	55.5	1650.7	12.3	39.1	17.2	3.8	13.3	8.0	2.1
Arithmetic mean	7183.9	24.3	1155.9	708.7	81.1	1906.7	16.3	42.9	46.9	8.7	14.9	20.3	3.2
Range	3985.6–11,263.5	10.0–58.5	433.4–2302.8	102.8–3443.9	6.5 -296.8	715.5–5582.1	1.8–177.3	16.5–117.7	5.8–460.2	0.6–53.0	0.6–38.9	3.0–116.8	0.2–12.1

**Table 14:** Main biomarkers of lung cancer identified in breath samples during the last decade.

Sakumura et al. [32]	hydrogen cyanide, methanol, acetonitrile, isoprene, 1-propanol
Oguma et al. [33]	cyclohexane and xylene
Callol-Sanchez et al. [34]	nonanoic acid
Schallschmidt et al. [25]	some aldehydes, 2-butanone and 1-butanol.
Rudnicka et al. [35]	propane, carbon disulfide, 2-propenal, ethylbenzene and isopropyl alcohol
Song et al. [36]	1-butanol and 3-hydroxy-2-butanone

## Data Availability

The data presented in this study are available on request from the corresponding author.
